# Construction of Mode of Action for Cadmium-Induced Renal Tubular Dysfunction Based on a Toxicity Pathway-Oriented Approach

**DOI:** 10.3389/fgene.2021.696892

**Published:** 2021-07-23

**Authors:** Yangchun Zhang, Ziqi Liu, Qianmei He, Fei Wu, Yongmei Xiao, Wen Chen, Yuan Jin, Dianke Yu, Qing Wang

**Affiliations:** ^1^Department of Toxicology, School of Public Health, Sun Yat-sen University, Guangzhou, China; ^2^Department of Toxicology, School of Public Health, Qingdao University, Qingdao, China

**Keywords:** cadmium, mode of action, renal tubular dysfunction, pathway analysis, weight of evidence

## Abstract

Although it is recognized that cadmium (Cd) causes renal tubular dysfunction, the mechanism of Cd-induced nephrotoxicity is not yet fully understood. Mode of action (MOA) is a developing tool for chemical risk assessment. To establish the mechanistic MOA of Cd-induced renal tubular dysfunction, the Comparative Toxicogenomics Database (CTD) was used to obtain genomics data of Cd-induced nephrotoxicity, and Ingenuity^®^ Pathway Analysis (IPA) software was applied for bioinformatics analysis. Based on the perturbed toxicity pathways during the process of Cd-induced nephrotoxicity, we established the MOA of Cd-induced renal tubular dysfunction and assessed its confidence with the tailored Bradford Hill criteria. Bioinformatics analysis showed that oxidative stress, DNA damage, cell cycle arrest, and cell death were the probable key events (KEs). Assessment of the overall MOA of Cd-induced renal tubular dysfunction indicated a moderate confidence, and there are still some evidence gaps to be filled by rational experimental designs.

## Introduction

Cadmium (Cd) is one of the most toxic metals and naturally exists in the environment. However, anthropogenic activities, including agricultural and industrial activities, contribute greatly to the pollution by Cd. The exposure sources in the general population include food, drinking water, smoking, and air. Dietary exposure is the main source in the general population, whereas occupational exposure in individuals working in alloy production, battery production, pigment production and use, plastics production, and smelting and refining mostly involves the respiratory tract (Faroon et al., 2012). Cd can accumulate in the human body due to its long biological half-life (10–30 years) (Kjellström and Nordberg, 1978; Nawrot et al., 2006; Amzal et al., 2009). After being absorbed, Cd is distributed throughout the body *via* blood circulation and accumulates mainly in the kidneys and liver—the major target organs of Cd toxicity. Epidemiological studies have demonstrated the association of Cd exposure and the increased risk of renal tubular impairment in both occupationally and environmentally exposed populations (Jarup et al., 2000; Akesson et al., 2005; Satarug et al., 2005; Teeyakasem et al., 2007; Wang et al., 2016). In blood, Cd is mostly found in the form of complexes consisting of low-molecular weight protein [mainly metallothionein (MT)]. These complexes readily pass through the glomeruli and are reabsorbed at the brush broader of renal tubules, where they subsequently accumulate and generate toxicity (Fujishiro et al., 2012, 2019). Cd accumulation in renal tubules can lead to tubular dysfunction characterized by Fanconi syndrome, which includes polyuria, proteinuria, and glucosuria (Johri et al., 2010). Although a large number of studies have focused on the mechanism of Cd-induced nephrotoxicity, which includes oxidative stress, mitochondrial dysfunction, genotoxicity, cell cycle arrest, and apoptosis, the data are still fragmented, and a systematic mode of action (MOA) remains to be established and applied to health risk assessment (Fujiwara et al., 2012; Prozialeck and Edwards, 2012; Matović et al., 2015; Song et al., 2016; Wang et al., 2017; Ge et al., 2019). Cd exposure has been recognized as an important public health issue since the beginning of the last century. The World Health Organization (WHO) and other institutes have issued a series of guidelines establishing limits for Cd exposure to protect the population from Cd-caused damage. However, it has been shown that damage, especially kidney impairment, occurs even under a “safe exposure level” (Satarug et al., 2010). Therefore, we need to improve the risk assessment of Cd and establish safer guidelines for exposure limits. MOA is a useful tool recommended by collaborative groups, including the International Programme on Chemical Safety and US Environmental Protection Agency, to identify critical precursor key events (KEs) and set the stage for human health risk assessment (Boobis et al., 2008).

MOA represents biologically plausible causal relationships and series of KEs, including molecular initiating event (MIE), which are essential for the occurrence of an effect [usually an adverse outcome (AO)]. These KEs can be empirically observed steps or their markers. They are essential but not necessarily sufficient in their own right for the occurrence of AO, and can occur on different biological levels, such as molecular level, cellular level, tissue/organ level, and individual level (Meek and Klaunig, 2010; Meek et al., 2014). With the indication of the robust MOA based on mechanistic data, early KEs and lower dose of chemical exposure related to the apical end points, which are traditionally observed in animal tests, can be effectively identified; thus, *in vitro* studies in human cells for characterizing early toxicity pathway perturbation with the employment of computational biology techniques can be applied for chemical risk assessment with less use of animals and reduced uncertainty in species, thereby conforming to the vision of “Toxicity Testing in the 21st Century” (National Research Council, 2007). To date, MOAs for toxicants, such as benzo[a]pyrene (BaP) and multi-walled carbon nanotubes have been established based on transcriptional genomics and applied to derive the points of departure (PODs) (Moffat et al., 2015; Labib et al., 2016), providing a new cost-effective paradigm for chemical risk assessment. However, compared with the construction of a MOA framework, the assessment of its confidence plays an equally important role and is pivotal to determine whether the putative MOA is robust enough to be further applied in chemical risk assessment.

Therefore, to provide a theoretical basis for Cd risk assessment, we constructed a putative MOA for Cd-induced renal tubular dysfunction based on bioinformatics analysis of genomics data from the Comparative Toxicogenomics Database (CTD). Weight of evidence analysis of the MOA revealed the potential data gaps in the present evidence, and further studies are needed to fill them.

## Materials and Methods

### Strategies to Obtain the Differentially Expressed Genes From the Comparative Toxicogenomics Database

**FIGURE 1:**
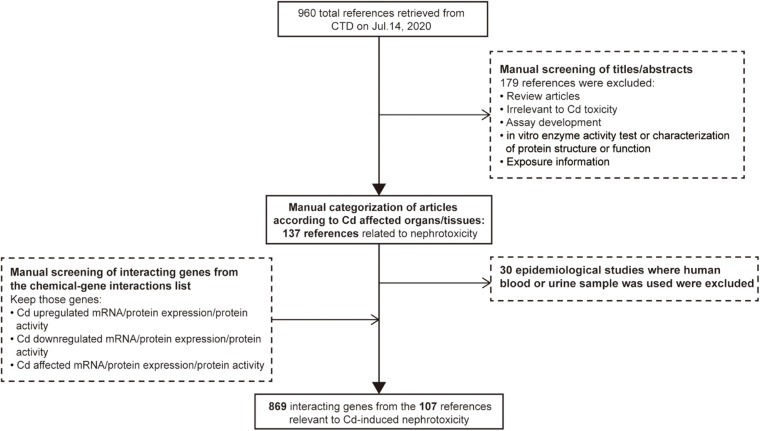
Workflow for selecting differentially expressed genes associated with cadmium-induced nephrotoxicity.

Comparative Toxicogenomics Database (CTD)^1^ is a public database containing the association information between chemical exposure, gene products, and diseases. It is manually organized by professional biocurators to promote the understanding of the effects of environmental chemicals on human health (Davis et al., 2011). The data of toxicogenomics, chemicals, diseases, and their interaction can help researchers to generate hypotheses about the molecular mechanisms of chemical-induced adverse health outcomes in support of experimental evidence (Davis et al., 2019). Therefore, CTD was employed to define the set of genes whose expression is influenced by Cd in the kidneys. The word “cadmium” was input into the chemical search box of CTD as a search term, and a total of 960 references were retrieved on July 14, 2020. Then, we manually screened the title or abstract of each reference. We excluded 149 references (review articles, studies irrelevant to Cd toxicity, assay development, *in vitro* enzyme activity tests, characterization of protein structure or function, and exposure characterization). The remaining references were then categorized according to Cd-affected organs/tissues. In addition, due to the lack of kidney specificity, we excluded epidemiological studies where human blood or urine samples had been used. The final set included 107 references (see Supplementary Sheet 1) related to Cd-induced nephrotoxicity and 869 genes whose expression or protein activity was increased/decreased/affected by Cd under the function model of chemical–gene interaction within each reference. The strategies of reference exclusion and differentially expressed gene selection are shown in Figure 1.

### Data Analysis With Ingenuity^®^ Pathway Analysis Software

Ingenuity^®^ Pathway Analysis (IPA) software (QIAGEN Inc.)^2^ was used to analyze the biological effects during Cd-induced nephrotoxicity by canonical pathway analysis and biofunction and disease analysis. Genes identified to be differentially expressed from CTD were input into IPA. Since CTD does not provide the detailed expression values or fold change values of the corresponding genes, we manually assigned the fold change value for those genes for the analysis in IPA. For transforming the qualitative expression alteration into a quantitative fold change value, we manually assigned the expression level of genes to 2, −2, or 0 based on the overall change in all of the included references. Genes with consistent change on either expression or protein activity (upregulated/downregulated/affected) in all the concerning references were assigned 2, −2, or 0, while the assignment of genes with contradictory expression change depended on the majority of cases. For example, SQSTM1 was involved in five references; in three of them, SQSTM1 was upregulated, while in two references, it was downregulated. Therefore, the expression level of SQSTM1 was assigned to 2. The 869 genes relevant to Cd-induced nephrotoxicity from 107 references in CTD were summarized in Supplementary Sheet 3. When setting parameters, we chose “human” as the specific species and the kidney as the target organ. We chose *p* < 0.05 as the threshold for significant perturbed pathways and excluded those that lacked z-score used to predict whether the pathway was activated or inhibited and the corresponding degree. The top 10 enriched pathways were selected to recognize the main effects relevant to the perturbed pathways.

### Construction and Assessment of Mode of Action

Mode of action (MOA) of a specific chemical consists of a series of events that are crucial to the occurrence of the AO; these KEs are measurable and sequential in chronological order (Meek and Klaunig, 2010). We recognized the biological effects of the top 10 significantly perturbed pathways as KEs. Their order was recognized according to the biological plausibility.

To initially confirm the reliability of the postulated MOA, the evolved Bradford Hill criteria were used to assess the evidence of the MOA, especially the causal relationship between the upstream KEs and downstream KEs. Five main aspects were considered in the evolved Bradford Hill criteria: biological plausibility, essentiality, empirical observation, consistency, and analogy. In the evaluation of essentiality, the focus was on the KEs, whereas in the evaluation of biological plausibility, empirical support, consistency, and analogy, the emphasis was on key event relationships (KERs). Biological plausibility required that the mechanistic relationships between KEs were consistent with the established biological knowledge. Essentiality was defined when the downstream KE would be prevented if the upstream KE was blocked. Empirical support included considerations about evidence for both dose–response and temporal concordance of KERs. Consistency referred to the consistency of empirical support for KERs across different species/strains/organs/test systems. Analogy support required that dependent alteration of KEs was observed after the exposure to the structurally related chemicals. Here, we put stress on the evaluation of KEs’ essentiality and empirical support for KERs because they have been weighted up to 80% in the assessment of MOA (Becker et al., 2017). The detailed defining questions and the relative confidence levels of evidence for the assessment of MOA were described in the published articles (Becker et al., 2015, 2017; OECD, 2018).

## Results

### Crucial Pathways and Key Events in Cadmium-Induced Nephrotoxicity

**FIGURE 2:**
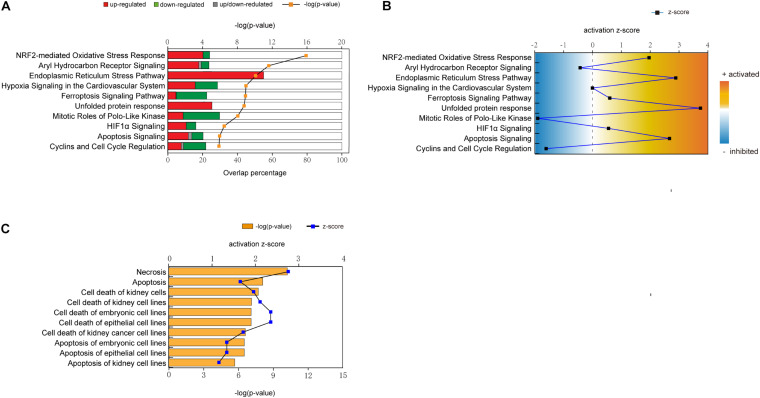
Crucial perturbed pathways, diseases, and biological functions involved in cadmium (Cd)-induced nephrotoxicity. A total of 869 genes (selection flow is shown in Figure 1) were entered into Ingenuity^®^ Pathway Analysis (IPA) together with the manually assigned fold change values to conduct core analysis. **(A)** Top 10 toxicity pathways involved in Cd-induced nephrotoxicity from canonical pathway analysis in IPA. Under the threshold *p* < 0.05 and rejecting the pathways without a z-score, we obtained the top 10 pathways in order of *p*-values from minimum to maximum. The red bars refer to the percentages of upregulated genes enriched in the corresponding pathways, the green bars refer to the percentage of downregulated genes enriched in the corresponding pathways, and the gray bars refer to the percentage of upregulated/downregulated genes enriched in the corresponding pathways. The overlap percentages were calculated as $\frac{\mathrm{number}\,\mathrm{of}\,\mathrm{enriched}\,\mathrm{genes}\,\mathrm{in}\,a\,\mathrm{pathway}}{\mathrm{total}\,\mathrm{number}\,\mathrm{of}\,\mathrm{genes}\,\mathrm{in}\,\mathrm{the}\,\mathrm{pathway}}$× 100%. **(B)** The activation z-score of the top 10 perturbed pathways. **(C)** Top 10 diseases and biological functions related to Cd from the module diseases and functions in IPA. After excluding the diseases and biological function terms without a predicted activation z-score, we ranked the diseases and biological functions in order of *p*-values. −log (*p*-value) and corresponding z-score of each entry are presented.

To identify the crucial toxicity pathways in Cd-induced nephrotoxicity, we entered the differentially expressed genes selected from CTD into IPA software to generate perturbed pathways. The summary of pathway perturbation and the corresponding enriched genes is presented in Supplementary Sheet 2. The top 10 toxicity pathways with the smallest *p*-value were as follows: nuclear factor erythroid 2-related factor 2 (Nrf2)-mediated oxidative stress response, aryl hydrocarbon receptor signaling, endoplasmic reticulum (ER) stress pathway, hypoxia signaling in the cardiovascular system, ferroptosis signaling pathway, unfolded protein response, mitotic roles of polo-like kinase, hypoxia-inducible factor-1-alpha (HIF1α) signaling, apoptosis signaling, and cyclins and cell cycle regulation (Figure 2A and Supplementary Figures 1–10). The positive value of z-score indicated the predicted activation of the pathway, while the negative value represented the predicted inhibition of the pathway, and the absolute value represented the degree of activation/inhibition. Hypoxia signaling in the cardiovascular system pathway, whose z score was 0, was neither activated nor inhibited (Figure 2B). Oxidative stress, DNA damage repair, cell cycle arrest, and apoptosis were the main biological effects triggered in the remaining nine pathways, which were either activated or inhibited (Table 1). The diseases and biofunctions analysis (Figure 2C) showed that cell death, especially apoptosis and necrosis, may be pivotal to the Cd-induced nephrotoxicity. This is concordant with the results from animal and cell experiments that showed that Cd could induce tubular necrosis *in vivo* and cell death *in vitro* (Fujiki et al., 2019; Hamidian et al., 2020).

**TABLE 1:** Biological effects triggered by the top 10 pathways*.

Pathway	Biological effects	Enriched genes
Nrf2-mediated oxidative stress response	Oxidative stress	*ATF4, CAT, DNAJA1, DNAJA4, DNAJB1, DNAJB4, DNAJB6, DNAJB9, EPHX1, FOS, FOSL1, FTH1, GCLC, GCLM, GSR, GSTA3, GSTA4, GSTM1, GSTO1, GSTP1, HMOX1, HSP90AA1, HSP90AB1, HSP90B1, HSPB8, JUN, JUNB, JUND, KEAP1, KRAS, MAF, MAFF, MAFG, MAPK1, MAPK3, MAPK8, NFE2L2, NQO1, PMF1, SOD1, SOD2, SQSTM1, TXNRD1, UBB*
Aryl hydrocarbon receptor signaling	DNA damage repair; cell cycle arrest; oxidative stress; cell death	*AIP, ALDH1A1, ALDH1L2, BAX, CCNA1, CCNA2, CDKN1A, CDKN1B, CHEK1, CTSD, FOS, GSTA3, GSTA4, GSTM1, GSTO1, GSTP1, HSP90AA1, HSP90AB1, HSP90B1, IL6, JUN, MAPK1, MAPK3, MAPK8, MDM2, MYC, NCOA7, NCOR2, NFE2L2, NQO1, TNF, TP53*
ER stress pathway	ER stress; apoptosis	*ATF4, ATF6, CALR, CASP3, CASP7, CASP9, DDIT3, EIF2S1, ERN1, HSP90B1, HSPA5, MAPK8*
Ferroptosis signaling pathway	Oxidative stress; cell death	*AIFM2, ANGPTL4, ARF1, ATF4, BECN1, BRAF, CDKN1A, FTH1, G3BP1, GCLC, GPX4, H2AX, HMOX1, KEAP1, KRAS, MAPK1, MAPK3, NFE2L2, PEBP1, SAT1, SLC7A11, SQSTM1, TFRC, TP53, TXNRD1*
Unfolded protein response	ER stress; apoptosis	*ATF4, ATF6, BCL2, CALR, DDIT3, DNAJA1, DNAJA4, DNAJB1, DNAJB4, DNAJB6, DNAJB9, ERN1, HSP90B1, HSPA1A/HSPA1B, HSPA5, HSPA8, HSPH1, MAPK8, NFE2L2, PPARG, PPP1R15A*
Mitotic roles of polo-like kinase	DNA damage repair; cell cycle arrest	*ANAPC11, CAPN1, CCNB1, CCNB2, CDC20, CDK1, FBXO5, HSP90AA1, HSP90AB1, HSP90B1, KIF11, KIF23, PLK1, PPP2CA, PPP2R1B, PRC1, PTTG1*
HIF1α signaling	Cell survival; ATP synthesis	*ADM, BRAF, CAMK2G, CDKN1A, EDN1, ELOB, HK2, HMOX1, HSP90AA1, HSPA1A/HSPA1B, HSPA5, HSPA8, IGF2, IL6, JUN, KRAS, LDHA, MAPK1, MAPK3, MDM2, MMP11, NOS2, RPS6, RPS6KB1, SAT1, SERPINE1, SLC2A3, SLC2A5, TP53*
Apoptosis signaling	DNA damage; apoptosis	*BAK1, BAX, BCL2, BCL2L1, BIRC2, BIRC3, CAPN1, CASP3, CASP7, CASP9, CDK1, CHUK, DIABLO, KRAS, MAPK1, MAPK3, MAPK8, TNF, TP53*
Cyclins and cell cycle regulation	Cell death	*CCNA1, CCNA2, CCNB1, CCNB2, CCNH, CDK1, CDKN1A, CDKN1B, CDKN2B, CDKN2C, E2F7, E2F8, FBXL5, PPP2CA, PPP2R1B, SKP2, TP53*

*Except for HS pathway because its z score = 0.

*ER, endoplasmic reticulum; HIF1α, hypoxia-inducible factor-1-alpha; Nrf2, nuclear factor erythroid 2-related factor 2.*

In this study, we used core analysis of IPA software through which we were able to simultaneously obtain the information of pathway enrichment, molecular interactions, and upstream prediction generated from the CTD gene set. CTD itself can also analyze pathway enrichment based on the Kyoto Encyclopedia of Genes and Genomes (KEGG) and REACTOME databases; thus, we also analyzed pathway enrichment by CTD with the support of KEGG. The results (Supplementary Figure 11) were similar to those from IPA, showing that apoptosis, cell cycle, and ER stress-related pathways were significantly perturbed.

In addition to the bioinformatics analysis, we also retrieved review articles to understand the established cytotoxicity mechanisms of Cd. It has been shown that Cd could cause oxidative stress not by directly increasing free radicals due to its non-redox nature but by indirectly generating reactive oxygen species (ROS) by interaction with Fenton metal, such as Fe and affecting the antioxidative defense system (Matović et al., 2015). Cd is not directly genotoxic because it lacks the capacity to generate DNA adducts, but it could induce indirect DNA damage by the increased ROS levels (Valverde et al., 2001). To avoid the wrong DNA replication entering into the next cell generation, the unrepaired DNA damage subsequently blocks cell cycle progression and initiates cell death (Takahashi et al., 2019). Thus, according to the main biological effects of top significant pathways and the biological relationship among them, key cellular events and the chronological order among them were recognized. Oxidative stress was shown to be the KE1, followed by DNA damage (KE2), cell cycle arrest (KE3), and cell death (KE4). The order is biologically plausible.

### Description of the Postulated Mode of Action

**FIGURE 3:**
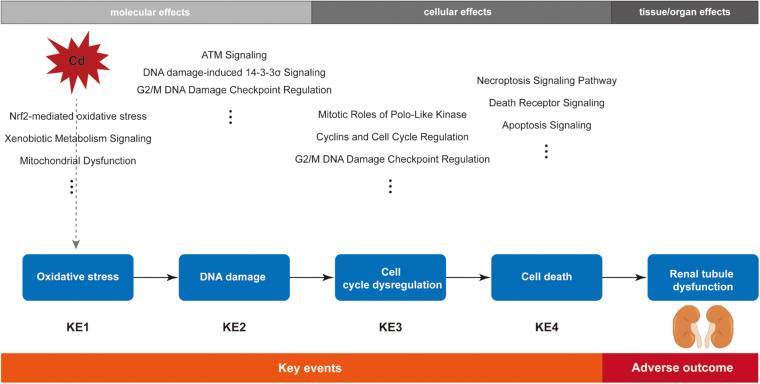
Schematic diagram of the postulated mode of action (MOA) for cadmium-induced renal tubular dysfunction.

Kidneys are the recognized target organs of chronic Cd exposure, especially the renal proximal tubules. Renal tubular dysfunction is an early clinical feature of renal damage caused by Cd, during which proteinuria (mainly low-molecular weight proteins, such as β2-microglobulin), glycosuria, amino aciduria, and polyuria have been observed in both humans and laboratory animals (Faroon et al., 2012). Furthermore, epidemiological studies have shown evidence of association between renal damage and Cd exposure (Akesson et al., 2005; Satarug et al., 2010). To date, researchers have explored various mechanisms of Cd-induced nephrotoxicity, but the MOA of Cd-induced renal tubular dysfunction has not been established yet. After integration of bioinformatics analysis and biological plausibility, we constructed the MOA of Cd-induced renal tubular dysfunction (Figure 3).

#### KE1: Oxidative Stress

In the results of canonical pathway analysis, in addition to the NRF2 pathway, which was 1 of the top 10 significant pathways, there are other pathways associated with oxidative stress, such as xenobiotic metabolism signaling pathway and mitochondrial dysfunction pathway (Supplementary Table 1), which may be involved in Cd-induced oxidative stress in the kidney.

Oxidative stress has been widely demonstrated to be a pivotal molecular mechanism in nephrotoxicity caused by acute or chronic Cd exposure (Renugadevi and Prabu, 2009; Wang et al., 2009; Nemmiche, 2017). Both *in vivo* and *in vitro* studies have provided plenty of evidence of oxidative stress playing an important role in Cd-induced nephrotoxicity, especially in tubular damage. Cd has been shown to enhance oxidative stress by triggering ROS production and inhibiting the antioxidant system in renal tubular epithelial cells; such oxidative damage and nephrotoxicity could be alleviated by various substances with antioxidant activity (Ognjanović et al., 2008; Wang et al., 2009, 2018, 2019; Nazima et al., 2015; Poontawee et al., 2016; Das et al., 2019; Joardar et al., 2019; Aqeel et al., 2020). Cd is a non-redox metal; thus, it is not able to directly produce free radicals, but it indirectly increases the generation of ROS and causes oxidative stress damage (Liu et al., 2008). Although Cd cannot directly generate free radicals, it can replace the iron component in some proteins, including ferritin and apoferritin, increasing free Fe^2+^ and ⋅OH generated from Fe-catalyzed Fenton action (Dewanjee et al., 2013; Matović et al., 2015). It has also been demonstrated that Cd could consume the antioxidant glutathione (GSH) and inhibit the activity of enzymes pivotal for maintaining GSH due to the high affinity between Cd^2+^ and -SH, such as glutathione reductase, glutathione peroxidase, and glutathione-S-transferase. Moreover, another potential mechanism by which Cd may decrease the activity of antioxidant enzymes is an interaction with divalent elements, such as manganese, zinc, and selenium; these elements function as cofactors and form the core active sites of antioxidant enzymes (e.g., superoxide dismutase, catalase, and glutathione reductase) (Cuypers et al., 2010; Matović et al., 2015). After chronic exposure to low-dose Cd, nephrotoxicity occurs with increased renal ROS level and decreased activity of antioxidant enzymes (Wang et al., 2013; Ansari et al., 2017; Liu et al., 2019). However, oxidative stress is a dynamic equilibrium process; there is an adaptive system to eliminate ROS, and oxidative damage would appear only when ROS was overloaded. Thus, we hypothesize that oxidative stress is an early event in Cd-induced nephrotoxicity, and it can be counterbalanced at least partly by the corresponding defense system.

#### KE2: DNA Damage

As shown in the result of canonical pathway (Supplementary Table 1), ATM signaling, role of BRCA1 in DNA damage response, DNA damage-induced 14-3-3σ signaling, and cell cycle: G2/M DNA damage checkpoint regulation are potential pathways involved in the DNA damage and DNA damage repair induced by Cd other than the pathways in the top 10 significant pathways.

DNA is a common target of free radicals generated from oxidative stress induced by exogenous chemicals, including Cd. Cd probably induces DNA damage not by direct interaction but rather indirectly through oxidative stress. Cd^2+^ alone did not lead to DNA damage in plasmid DNA; however, it was able to cause DNA strand breaks in the presence of hydrogen peroxide in a dose-dependent manner due to the production of hydroxyl radicals (Badisa et al., 2007). Likewise, Valverde et al. (2001) reported that no direct induction of DNA damage by Cd was detected, and the genotoxicity probably involved an indirect interaction due to oxidative stress. The oxidative stress-associated DNA damage was found in both *in vitro* renal tubule cells and kidney tissues from *in vivo* experiments under Cd treatment (Saplakoğlu et al., 1997; Chater et al., 2008). Induction of DNA damage subsequently activates the DNA repair system to prevent the wrong DNA replication from entering the next cell generation. Cell cycle checkpoints and cell cycle arrest ensure adequate time for the DNA repair process (Nyberg et al., 2002). The cell fate depends on whether the DNA damage is repaired. If the DNA damage is successfully repaired, the cell cycle progression continues. If the repair fails, cell death is initiated (Harper and Elledge, 2007; Ciccia and Elledge, 2010).

#### KE3: Cell Cycle Arrest

The results of pathway analysis (Supplementary Table 1) showed that the significant pathways associated with cell cycle progression had negative z scores, while pathways associated with cell cycle arrest had positive z scores, indicating that the cell cycle may be arrested by Cd in the kidney. Pathways, such as ATM signaling and cell cycle: G2/M DNA damage checkpoint regulation were predicted to be activated (z score > 2), which indicated that these pathways may play critical roles in Cd-induced cell cycle dysregulation in the kidney.

Bork et al. (2010) found that the DNA damage caused by Cd could trigger the G2/M cell cycle arrest in kidney proximal tubule cells. Luo et al. (2020) reported that different concentrations of Cd led to different cell fates, depending on the extent of DNA damage in NRK-52E cells. At a low concentration (2.5 μM), Cd induced G0/G1 cell cycle arrest, and eventually the cells survived from slight damage; in contrast, 10 μM Cd caused severe DNA damage, leading to S phase cell cycle arrest and ultimately to cell death. These findings suggested that Cd could inhibit DNA repair and lead to cell death.

#### KE4: Cell Death

Previous studies showed that Cd could induce renal tubular cell death *via* oxidative stress and unrepaired DNA damage (Suliman Al-Gebaly, 2017; Zhuang et al., 2019). Different forms of cell death induced by Cd have been reported. Most studies have revealed that cell apoptosis and necrosis are the main forms of Cd-induced cell death, and a few studies indicated that pyroptosis might also be one of the forms of Cd-induced cell death (Fujiwara et al., 2012; Chou et al., 2019; Fujiki et al., 2019). It has been recognized that renal tubule necrosis occurs when animals are exposed to a high dose of Cd for a long time. However, before necrosis, renal tubular dysfunction with cell apoptosis has been observed (Hamada et al., 1991; Tanimoto et al., 1993; Yan et al., 1997). Therefore, cell apoptosis, rather than necrosis, is more likely to be the crucial event in the early stage of renal tubular dysfunction before obvious kidney injury. The following three pathways have been suggested to be the probable apoptotic pathways: ER stress mediated by unfolded protein reaction, caspase-dependent/independent pathway, and p53 signaling pathway (Fujiwara et al., 2012). These suggestions are consistent with the results of our bioinformatics analysis that showed that the ER stress-related pathways (ER stress pathway and unfolded protein in response pathway) were significantly activated and the apoptotic genes, such as CASPs, BAX/BCL2, and TP53, were involved in apoptosis-associated pathways. In addition, significant pathways, such as apoptosis signaling pathway, death receptor signaling, and MYC-mediated apoptosis signaling were predicted to be activated (z score > 2) (Supplementary Table 1).

#### Adverse Outcome: Renal Tubular Dysfunction

Cd is a recognized toxicant that can generate toxicity in renal tubules and cause renal injury. *In vivo* studies have demonstrated that chronic Cd exposure leads to renal tubular dysfunction characterized as low-molecular weight proteinuria and histopathological changes of renal tubules (e.g., tubular degeneration, brush border atrophy, and epithelial cell fragmentation release into lumen) (Brzóska et al., 2003; Asar et al., 2004; Wu et al., 2012). Renal proximal tubule is the site where Cd tends to accumulate, and its dysfunction coincides with cell apoptosis prior to obvious tubular necrosis after Cd intoxication (Friberg, 1984; Prozialeck and Edwards, 2012). Therefore, dysfunction of reabsorption seems to be the early stage of Cd-induced renal tubular injury. However, in most experiments, renal tubular epithelial cell apoptosis and renal tubular dysfunction have been observed simultaneously; that is, there is a lack of evidence about dose/time-dependent response to explain the causal relationship between cell apoptosis and renal tubular dysfunction. In addition to *in vivo* experiments, epidemiological studies also showed associations between Cd exposure and renal tubular dysfunction among populations (Akesson et al., 2005; Satarug et al., 2010).

### Assessment of Confidence of the Proposed Mode of Action Based on Tailored Bradford Hill Criteria

According to the guidance document released by the Organization for Economic Co-operation and Development (OECD, 2018), assessment of the essentiality of all KEs and overall assessment for KERs with tailored Bradford Hill criteria are required for the overall assessment of MOA. Herein, we focused on assessing essentiality of each KE and the empirical support for KERs using the defining questions described in previous studies (Becker et al., 2015, 2017; OECD, 2018). The latter included concordance in dose–response, temporal, and incidence relationships between KEs.

#### Essentiality of Key Events

To explore the confidence of supporting data for the essentiality of KEs in the MOA, we assembled evidence and summarized it in Table 2.

**TABLE 2:** Essentiality of KEs and the responding confidence.

KE1: oxidative stress
The confidence is strong. There is bulky supporting evidence on the essentiality of this KE. Substances possessing antioxidant capacity successfully ameliorated the nephrotoxicity induced by Cd Renugadevi and Prabu, 2009; Nazima et al., 2015; Das et al., 2019; Joardar et al., 2019; Guan et al., 2020.
**KE2: DNA damage**
The confidence is moderate. Biological plausibility provides strong support for the essentiality of this event, but there is inadequate supporting evidence on observation of end point effects under the specific DNA damage inhibitor.
**KE3: cell cycle arrest**
The confidence is moderate. Relieving G2/M transition block decreased cell cycle arrest induced by DNA damage and increased cell apoptosis Bork et al., 2010.
**KE4: cell death**
The confidence is weak. There is inadequate experimental evidence for the essentiality of cell death for Cd-induced renal tubular dysfunction.

*Cd, cadmium; KE, key event.*

#### Empirical Support for Key Event Relationships

Since MOA consists of a series of sequential, measurable KEs essential to AO, in addition to the assessment of essentiality of each KE, evidence concordance of all KERs is also required. Thus, we assessed the evidence concordance including the dose–response and the temporal relationship between KEs.

The existing knowledge has provided biological plausibility of KERs in the MOA that we constructed. ROS indirectly produced by Cd^2+^ could attack DNA and induce DNA damage by breaking DNA strands. DNA damage accumulation challenges DNA repair and subsequently blocks cell cycle progression and initiates cell death to avoid wrong replication entering into divided cells (Ciccia and Elledge, 2010). However, evidence supporting the concordance of KERs from either dose–response or temporal consistency is not as strong as biological plausibility. In most studies, these KEs were first observed at the same concentrations or time points as shown in Table 3. This may be attributed to the relatively high concentration of Cd, at which the tested cells or animals would undergo more severe damage and activate both the early and the late KEs. In addition, inability to select more intervals for the analysis of time-dependent effects increases the difficulty to figure out the temporal sequence of KEs. Noticeably, a study (Ge et al., 2018) of Cd-induced cytotoxicity on HK-2 cells under a low concentration of 1 μM showed that during the 12-day exposure period, oxidative stress (KE1) was first observed on day 2, while the early cell apoptosis (KE3) was significantly increased from day 10. The results indeed supported the temporal relationship of KE1 and KE3; however, the significant increase of ROS only occurred on days 2 and 4, suggesting that it is not the transient activation of oxidative stress, but rather another oxidative stress-related mechanism, that directly leads to apoptosis. Unfortunately, only a few studies showed obvious dose–response and temporal concordance between either adjacent or nonadjacent KEs (Fasanya-Odewumi et al., 1998; Nemmiche and Guiraud, 2016; Ge et al., 2018), suggesting that the supporting evidence is not strong and further verification needs to be designed.

**TABLE 3:** Dose–response and temporal concordance of KERs.

Model tested	Time point	Concentration tested	KE1	KE2	KE3	AO	Reference
Primary rat kidney proximal tubule cells	1 h	50, 100 μM	50 μM^a^	50 μM	N/A	N/A	Bork et al., 2010
Primary rat kidney proximal tubule cells	3 h	50, 100 μM	50 μM	50 μM	N/A	N/A	Bork et al., 2010
Primary rat kidney proximal tubule cells	6 h	50, 100 μM	50 μM	50 μM	50 μM	N/A	Bork et al., 2010
Primary duck renal tubular epithelial cells	12 h	1.25, 2.5, 5 μM	1.25 μM	N/A	1.25 μM	N/A	Zhuang et al., 2019
HK-2 cells	1, 2, 4, 6, 8, 10, 12 days	1 μM	2 days	N/A	10 days	N/A	Ge et al., 2018
BJAB cells	24 h	5, 10, 20, 40 μM	5 μM	10 μM	N/A	N/A	Nemmiche and Guiraud, 2016
NRK52E cells	18 h	5, 10, 20, 30, 50 μM	N/A	20 μM	20 μM	N/A	So and Oh, 2016
NRK52E cells	1, 3, 6, 12, 24 h	35 μM	N/A	12 h	12 h	N/A	So and Oh, 2016
ZFL cells	24 h	5.45, 27.27, 54.55 μM	5.45 μM	5.45 μM	N/A	N/A	Morozesk et al., 2020
NRK52E cells	48 h	1, 10 μM	10 μM	N/A	10 μM	N/A	Jimi et al., 2004
Rats	14, 21, 28, 35, 42 days	14, 28 mg/kg	N/A	14 d	N/A	21 d	Fasanya-Odewumi et al., 1998

*^*a*^The minimum dose or the first time point when the corresponding events occur in the specific experiment design.*

*AO, adverse outcome; KE, key event; KER, key event relationship.*

## Discussion

In the present study, we used the toxicogenomics data from CTD to understand the perturbed pathways involved in Cd-induced nephrotoxicity, and we initially constructed the MOA of Cd-induced renal tubular dysfunction. Indeed, the genomics alteration induced by Cd treatment provided us with the bioinformatics indication of key cellular events, but the genomics data from CTD were not able to identify the dose–response relationship and temporal sequence between those KEs. Despite such flaws, this method could make use of the genomics information derived from the references collected by the frequently updated CTD, which would save us a lot of time on literature retrieval and review and reduce the reference omitting due to manual retrieval.

In the postulated MOA of Cd-induced renal tubular dysfunction, we could not recognize the MIE based on the bioinformatics analysis. Cd has a high affinity for the -SH-rich protein MT, which can respond to Cd stimulation. However, free Cd^2+^ instead of Cd–MT is the toxic form of Cd (Liu et al., 1998, 2000). Most Cd is absorbed at renal tubular epithelium in the form of Cd–MT complex by endocytosis, and the acidic environment of endosome or lysosome promotes the dissociation of Cd^2+^ from MT (Sabolić et al., 2010). Increased dissociation of Cd^2+^ from Cd–MT could be the MIE of Cd-induced renal tubular dysfunction. However, the MIE has not yet been experimentally verified, which requires detecting methods to distinguish the intracellular free Cd^2+^ from combined Cd. Methods, such as atomic absorption spectrometry (AAS) and inductively coupled plasma mass spectrometry (ICP-MS) have widely been used in the determination of Cd content in biological samples, but they failed to differentiate the intracellular ionic Cd (Jablonska et al., 2017; Wu et al., 2019). *In vitro* models need to be developed with the aim to measure the transition of Cd from the combined form to free ion and explore how the free Cd^2+^ affects the antioxidant system to trigger the downstream KEs.

To generate the postulated MOA of Cd-induced renal tubular dysfunction, we used a strategy of data mining from CTD to perform the pathway analysis with those genes whose mRNA and protein expression levels were affected by Cd. Actually, we also analyzed the perturbed pathways involved in Cd toxicity in liver and breast (see Supplementary Table 2) with the strategies similar to those depicted in the preceding text. In our MOA framework, KEs were not specific for nephrotoxicity. Oxidative stress, one of the reported mechanisms of Cd toxicity, was also involved in hepatotoxicity caused by Cd due to significant perturbation of Nrf2 signaling pathway. Similarly, cell cycle arrest-related pathway was activated by Cd in the breast, and apoptosis signaling was activated by Cd in the liver and breast. Thus, MIE is probably what determines the specificity of the MOA for Cd-induced nephrotoxicity. Regretfully, in our research, we failed to recognize the MIE for Cd-induced nephrotoxicity due to the lack of specific dose–effect and time–effect information from the CTD dataset to help determine the earliest event and no suggestive clue on structural information to predict the direct interaction between Cd and the potential initiating molecule.

We used the Hill Bradford criteria to assess the weight of evidence of the postulated MOA. The biological plausibility supporting the MOA was strong; however, for the essentiality and empirical support, the confidence was weaker. The essentiality of KE1 was well-supported by evidence considering that blockages of this event successfully reversed the downstream KEs. However, the essentiality of later KEs was insufficient, as there were data gaps for direct evidence (e.g., inhibitors of DNA damage/promoters for DNA repair block cell death induced by Cd in kidney tissue or *in vitro* renal tubular cells). Furthermore, not all KERs were well-supported by empirical evidence; indeed, in most cases, several KEs were observed simultaneously, especially the adjacent KEs. Thus, to increase the confidence of each KE, *in vivo* and *in vitro* studies involved in Cd-induced renal tubular dysfunction should set lower Cd concentrations and smaller time intervals to identify the dose–response and temporal sequence between each KE. Benchmark dose (BMD) analysis could help to verify the dose–response or temporal sequences of KEs.

In addition, in view of the weaker confidence for KE3 and KE4, we considered some alternative pathways in the MOA of Cd-induced kidney damage. ER stress, a probable mechanism of Cd toxicity, was also shown to be a potential KE based on the toxicity pathway analysis by IPA. In the top 10 perturbed pathways, ER stress pathway and unfolded protein response pathway were significantly enriched and were predicted to be activated. Furthermore, previous studies have reported the potential role of ER stress in Cd-induced nephrotoxicity. It has been reported that ER stress can regulate the apoptosis induced by Cd exposure (Ge et al., 2018; Li et al., 2020). However, the relationships between ER stress and the KEs (KE1, KE2, and KE3) in our postulated MOA were not clear. Thus, it is interesting to explore the crosstalk between the potential KEs and construct a comprehensive MOA.

MOA was recognized as a useful tool for chemical risk assessment. The use of quantitative transcriptomic data to determine BMDs based on a chemical’s MOA was demonstrated to be comparable to those derived from adverse apical effects (Hester et al., 2015; Webster et al., 2015; Labib et al., 2016). The KEs in the MOA could be alternative toxic end points for the traditional apical end points used in risk assessment, and more sensitive PODs may be obtained in this way. In addition, the pathway-oriented KE will help us to develop new strategies in future chemical toxicity tests. For example, key molecules in pathways associated with KEs can serve as biomarkers or new indications for toxicity assays. Thus, the MOA we constructed provides a new insight for Cd risk assessment.

In summary, based on the pathway-oriented approach, here we constructed the MOA framework for Cd-induced renal tubular dysfunction. Oxidative stress response was the KE1, followed by DNA damage, cell cycle arrest, and cell death as the later KEs, and eventually renal tubular dysfunction was the AO. Weight of evidence analysis for our MOA suggested that data gaps still exist, especially in terms of MIE. The MOA has to be verified in further studies before it can be used for chemical risk assessment.

## Data Availability Statement

The original contributions presented in the study are included in the article/Supplementary Material, further inquiries can be directed to the corresponding author/s.

## Author Contributions

YZ wrote the main manuscript text, contributed to data collection and bioinformatics analysis, and prepared the figures. QW proposed and organized the study, contributed to data interpretation, and revised the manuscript. YJ, DY, YX, and WC contributed to the study design. ZL, QH, and FW contributed to the data analysis. All authors reviewed the manuscript.

## Conflict of Interest

The authors declare that the research was conducted in the absence of any commercial or financial relationships that could be construed as a potential conflict of interest.

## Publisher’s Note

All claims expressed in this article are solely those of the authors and do not necessarily represent those of their affiliated organizations, or those of the publisher, the editors and the reviewers. Any product that may be evaluated in this article, or claim that may be made by its manufacturer, is not guaranteed or endorsed by the publisher.

## Footnotes

1 http://ctdbase.org

2 https://www.qiagen.com/us/products/informatics-and-data/interpretation-content-databases/ingenuity-pathway-analysis/#orderinginformation
