# Supplementary material for: Construction of Mode of Action for Cadmium-Induced Renal Tubular Dysfunction Based on a Toxicity Pathway-Oriented Approach
Source: Front Genet. 2021 Jul 23;12:696892. doi: 10.3389/fgene.2021.696892 (PMC8343180; doi:10.3389/fgene.2021.696892)
Supplement: Supplementary file 2 [file Data_Sheet_2.docx]

# Supplementary Tables

**Table S1.** Pathways related to KEs in cadmium-induced renal injury

| Ingenuity Canonical Pathways | -log(p-value) | Ratio | Z score | Molecules |
| --- | --- | --- | --- | --- |
| **KE1: oxidative stress** | | | | |
| NRF2-mediated Oxidative Stress Response | 13.1 | 0.226 | 1.961 | ATF4, CAT, DNAJA1, DNAJA4, DNAJB1, DNAJB4, DNAJB6, DNAJB9, EPHX1, FOS, FOSL1, FTH1, GCLC, GCLM, GSK3B, GSR, GSTA3, GSTA4, GSTM1, GSTO1, HMOX1, HSPB8, JUN, JUNB, JUND, KEAP1, KRAS, MAF, MAFF, MAFG, NFE2L2, NQO1, PMF1, SOD1, SOD2, SQSTM1, TXNRD1, UBB |
| Superoxide Radicals Degradation | 3.22 | 0.5 | 1 | CAT, NQO1, SOD1, SOD2 |
| Xenobiotic Metabolism Signaling | 6.97 | 0.151 | NA | AIP, ALDH1A1, ALDH1L2, CAMK2G, CAT, ESD, GCLC, GSTA3, GSTA4, GSTM1, GSTO1, GSTP1, HMOX1, HSP90AA1, HSP90AB1, HSP90B1, IL6, KEAP1, KRAS, MAF, MAOA, MAOB, MAPK1, MAPK11, MAPK3, MAPK8, NCOR2, NFE2L2, NOS2, NQO1, PPP2CA, PPP2R1B, SULT2B1, SUMO1, TNF |
| Mitochondrial Dysfunction | 3.91 | 0.139 | NA | ATP5F1A, ATP5MC1, ATP5PD, BCL2, CASP3, CASP9, CAT, COX5B, COX6B2, COX8A, GPX4, GSR, HSD17B10, LRRK2, MAOA, MAOB, MAPK8, NDUFV1, PDHA1, SOD2, XDH |
| **KE2: DNA damage** | | | | |
| Role of BRCA1 in DNA Damage Response | 1.69 | 0.125 | 0 | BABAM2, CDKN1A, CHEK1, E2F7, E2F8, FANCF, GADD45A, PLK1, TP53 |
| ATM Signaling | 4.71 | 0.188 | 2.138 | ATF4, CCNB1, CCNB2, CDK1, CDKN1A, CHEK1, CREB1, GADD45A, H2AX, JUN, MAPK11, MAPK8, MDM2, PPP2CA, PPP2R1B, TP53 |
| DNA damage-induced 14-3-3σ Signaling | 2.57 | 0.278 | NA | CCNB1, CCNB2, CDK1, RAD1, TP53 |
| Cell Cycle: G2/M DNA Damage Checkpoint Regulation | 5.76 | 0.271 | 3.051 | AURKA, CCNB1, CCNB2, CDK1, CDKN1A, CHEK1, FBXL5, GADD45A, MDM2, PLK1, SKP2, TP53, YWHAZ |
| **KE3: cell cycle arrest** | | | | |
| ATM Signaling | 4.71 | 0.188 | 2.138 | ATF4, CCNB1, CCNB2, CDK1, CDKN1A, CHEK1, CREB1, GADD45A, H2AX, JUN, MAPK11, MAPK8, MDM2, PPP2CA, PPP2R1B, TP53 |
| Mitotic Roles of Polo-Like Kinase | 8.04 | 0.298 | -1.897 | ANAPC11, CAPN1, CCNB1, CCNB2, CDC20, CDK1, FBXO5, HSP90AA1, HSP90AB1, HSP90B1, KIF11, KIF23, PLK1, PPP2CA, PPP2R1B, PRC1, PTTG1 |
| Cyclins and Cell Cycle Regulation | 5.88 | 0.218 | -1.604 | CCNA1, CCNA2, CCNB1, CCNB2, CCNH, CDK1, CDKN1A, CDKN1B, CDKN2B, CDKN2C, E2F7, E2F8, FBXL5, PPP2CA, PPP2R1B, SKP2, TP53 |
| PTEN Signaling | 3.7 | 0.142 | -0.728 | BCL2, BCL2L1, CASP3, CASP9, CDKN1A, CDKN1B, CHUK, FGFR1, FGFR2, IGF2R, ILK, INPP5J, INPP5K, KRAS, MAGI1, MAPK1, MAPK3, RPS6KB1, TGFBR1 |
| Cell Cycle: G1/S Checkpoint Regulation | 3.53 | 0.182 | 0.333 | CDKN1A, CDKN1B, CDKN2B, CDKN2C, E2F7, E2F8, FBXL5, MDM2, MYC, NRG1, SKP2, TP53 |
| Role of CHK Proteins in Cell Cycle Checkpoint Control | 3.73 | 0.204 | 0.632 | ATMIN, CDK1, CDKN1A, CHEK1, E2F7, E2F8, PLK1, PPP2CA, PPP2R1B, RAD1, TP53 |
| Cell Cycle: G2/M DNA Damage Checkpoint Regulation | 5.76 | 0.271 | 3.051 | AURKA, CCNB1, CCNB2, CDK1, CDKN1A, CHEK1, FBXL5, GADD45A, MDM2, PLK1, SKP2, TP53, YWHAZ |
| **KE4: cell death** | | | | |
| Necroptosis Signaling Pathway | 2.26 | 0.118 | 0 | BIRC2, BIRC3, CAMK2G, CAPN1, CHUK, MDM2, PELI1, PPID, PPIF, SLC25A4, SLC25A5, TIMM50, TNF, TNFSF10, TP53 |
| Endoplasmic Reticulum Stress Pathway | 10.1 | 0.6 | 2.887 | ATF4, ATF6, CALR, CASP3, CASP7, CASP9, DDIT3, EIF2S1, ERN1, HSP90B1, HSPA5, MAPK8 |
| Unfolded protein response | 8.77 | 0.266 | 3.742 | ATF4, ATF6, BCL2, CALR, DDIT3, DNAJA1, DNAJA4, DNAJB1, DNAJB4, DNAJB6, DNAJB9, ERN1, HSP90B1, HSPA1A/HSPA1B, HSPA5, HSPA8, HSPH1, MAPK8, NFE2L2, PPARG, PPP1R15A |
| Death Receptor Signaling | 2.28 | 0.132 | 2.111 | BCL2, BIRC2, BIRC3, CASP3, CASP7, CASP9, CHUK, DIABLO, MAPK8, TNF, TNFSF10, TNFSF15 |
| MYC Mediated Apoptosis Signaling | 5.65 | 0.265 | 2.111 | ADRB1, BAX, BCL2, BCL2L1, CASP3, CASP9, CHUK, MDM2, MYC, PMAIP1, PRKAR1A, TNF, TP53 |
| Apoptosis Signaling | 5.96 | 0.202 | 2.668 | BAK1, BAX, BCL2, BCL2L1, BIRC2, BIRC3, CAPN1, CASP3, CASP7, CASP9, CDK1, CHUK, DIABLO, KRAS, MAPK1, MAPK3, MAPK8, TNF, TP53 |
| PTEN Signaling | 3.7 | 0.142 | 0.728 | BCL2, BCL2L1, CASP3, CASP9, CDKN1A, CDKN1B, CHUK, FGFR1, FGFR2, IGF2R, ILK, INPP5J, INPP5K, KRAS, MAGI1, MAPK1, MAPK3, RPS6KB1, TGFBR1 |

**Table S2.** Top 10 toxicity pathways perturbed by Cd in liver and breast

| Perturbed Pathways | -log(p-value) | Ratio | z-score |
| --- | --- | --- | --- |
| **Liver (112 references; 293 genes)** | | | |
| Sirtuin Signaling Pathway | 18.9 | 0.171 | 0 |
| Aryl Hydrocarbon Receptor Signaling | 16.4 | 0.216 | 0.632 |
| NRF2-mediated Oxidative Stress Response | 14.7 | 0.184 | 1.89 |
| Hepatic Fibrosis Signaling Pathway | 14.5 | 0.128 | 2.711 |
| HMGB1 Signaling | 14.4 | 0.214 | 2.887 |
| Xenobiotic Metabolism General Signaling Pathway | 13.2 | 0.202 | 0.632 |
| Xenobiotic Metabolism CAR Signaling Pathway | 12.6 | 0.177 | 2.121 |
| Senescence Pathway | 12 | 0.141 | 0.577 |
| Xenobiotic Metabolism AHR Signaling Pathway | 11.7 | 0.25 | 1.732 |
| Apoptosis Signaling | 10.6 | 0.213 | 2.121 |
| **Breast (24 references; 880 genes)** | | | |
| Aryl Hydrocarbon Receptor Signaling | 4.57 | 0.13 | 1.667 |
| Cell Cycle: G2/M DNA Damage Checkpoint Regulation | 3.15 | 0.167 | 0.707 |
| Induction of Apoptosis by HIV1 | 2.66 | 0.14 | 1.342 |
| GP6 Signaling Pathway | 2.59 | 0.118 | -1.897 |
| Myc Mediated Apoptosis Signaling | 2.53 | 0.149 | 2 |
| Endothelin-1 Signaling | 2.47 | 0.0977 | 2.309 |
| Endocannabinoid Cancer Inhibition Pathway | 2.46 | 0.102 | -0.905 |
| p53 Signaling | 2.45 | 0.112 | 0.707 |
| Osteoarthritis Pathway | 2.44 | 0.0904 | 0.905 |
| Agrin Interactions at Neuromuscular Junction | 2.38 | 0.127 | 0.378 |
