# Supplementary material for: Construction of Mode of Action for Cadmium-Induced Renal Tubular Dysfunction Based on a Toxicity Pathway-Oriented Approach
Source: Front Genet. 2021 Jul 23;12:696892. doi: 10.3389/fgene.2021.696892 (PMC8343180; doi:10.3389/fgene.2021.696892)
Supplement: Supplementary file 3 [file Data_Sheet_3.pdf]

Supplementary Figures

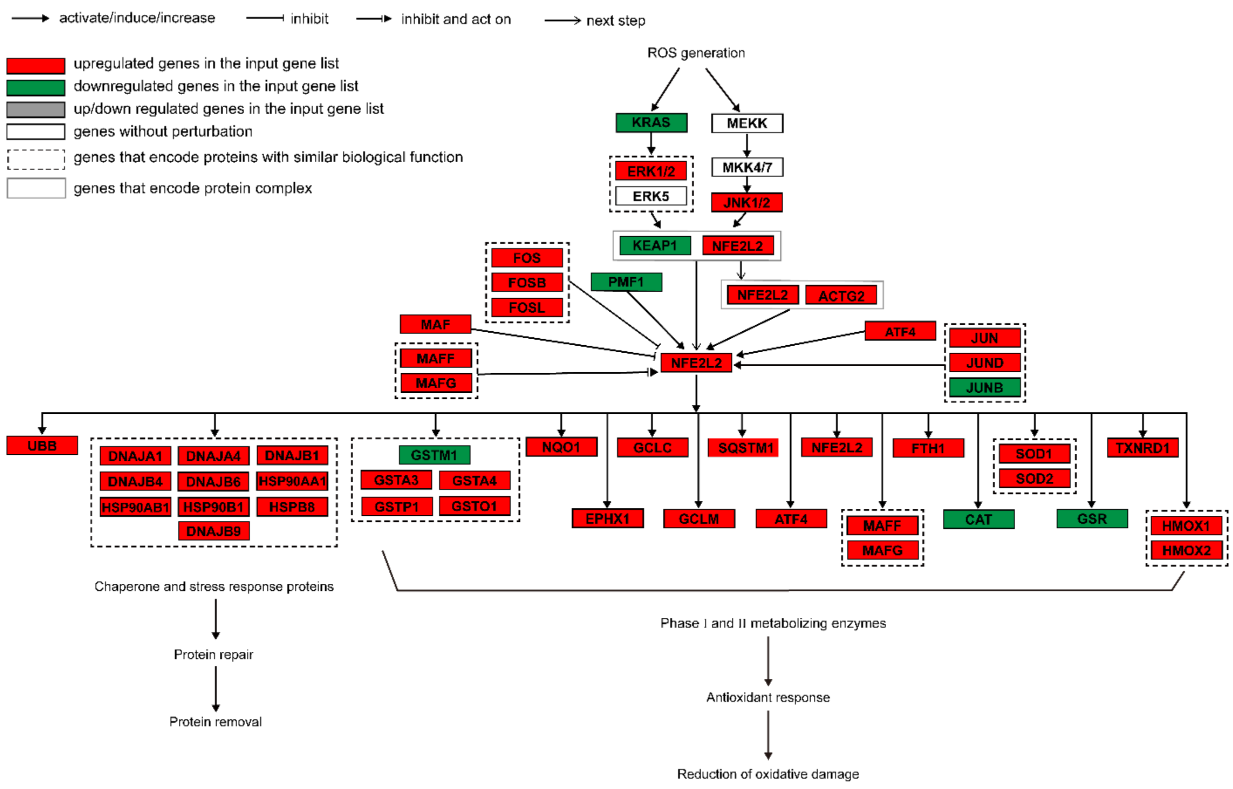

Supplementary Figure 1. NRF2-mediated oxidative stress response.

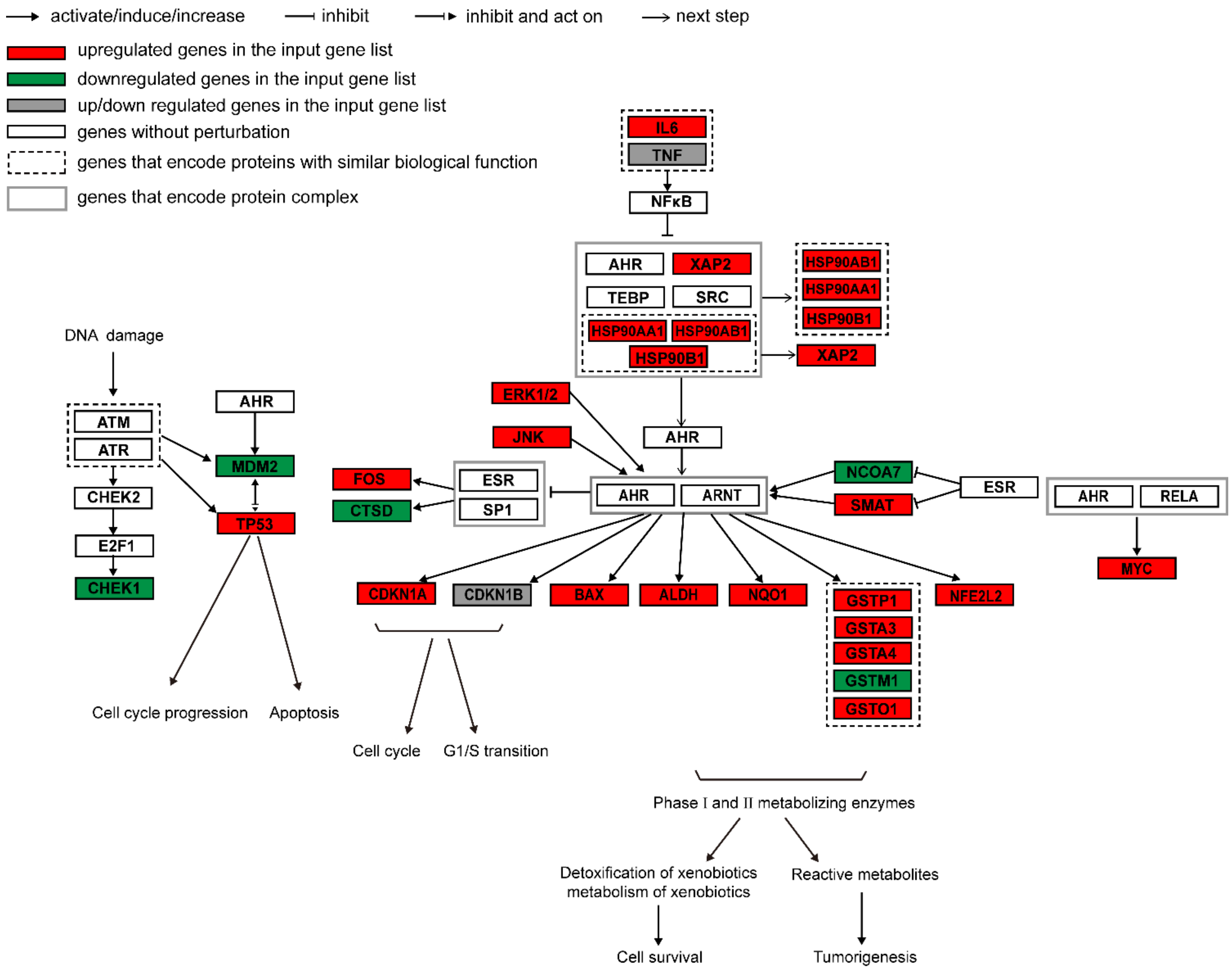

Supplementary Figure 2. Aryl hydrocarbon receptor signaling.

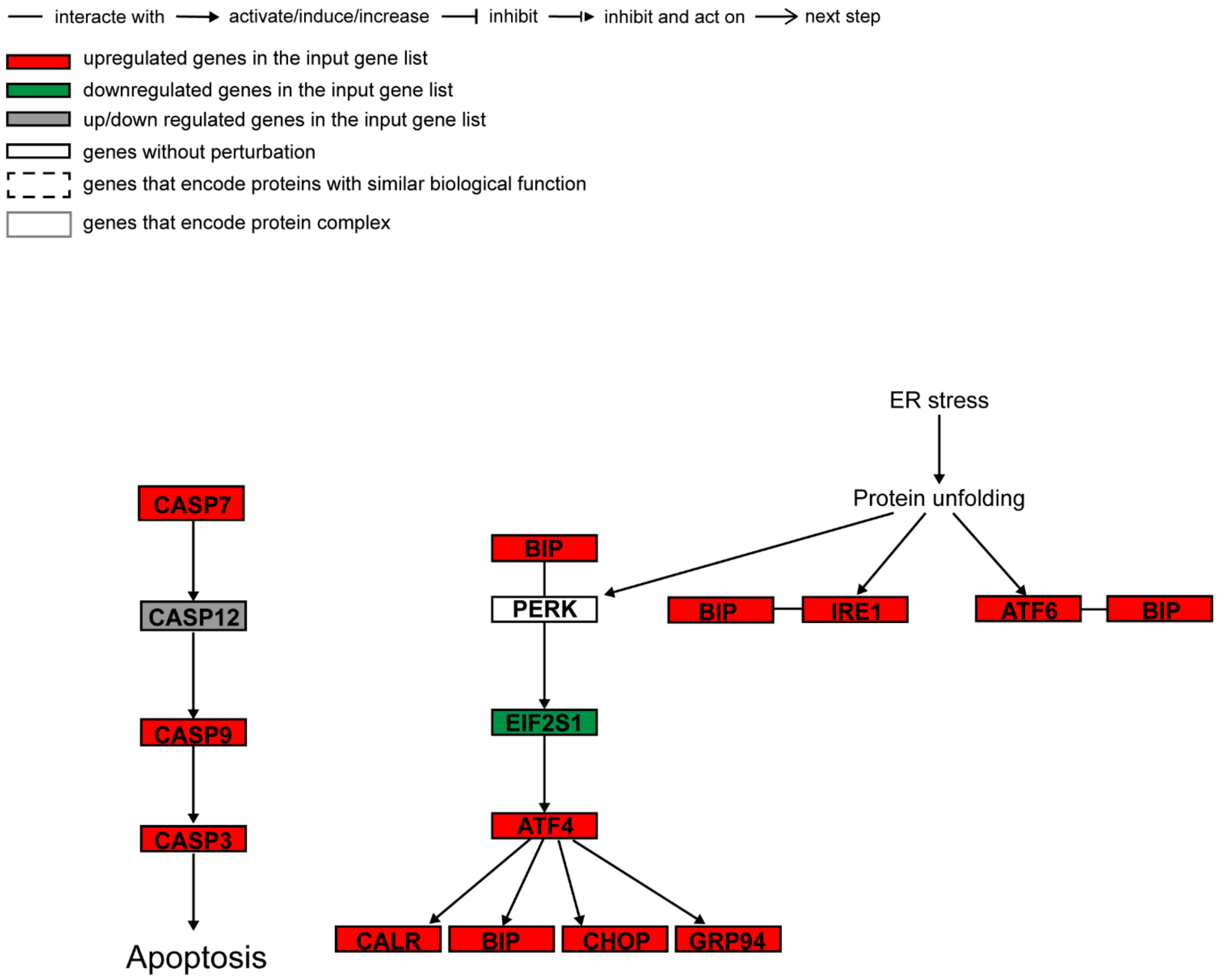

Supplementary Figure 3. Endoplasmic reticulum stress pathway

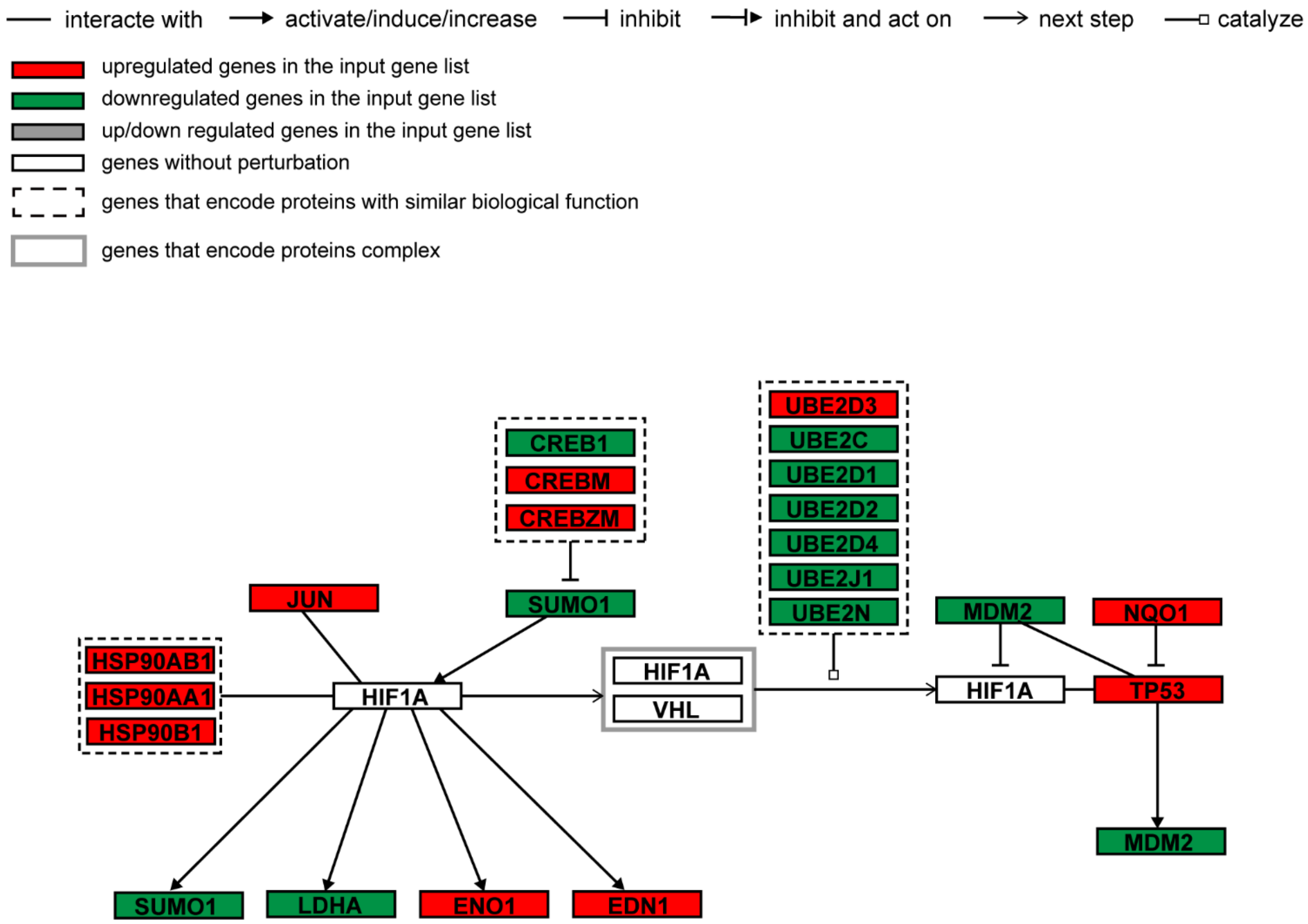

Supplementary Figure 4. Hypoxia signaling in the cardiovascular system.

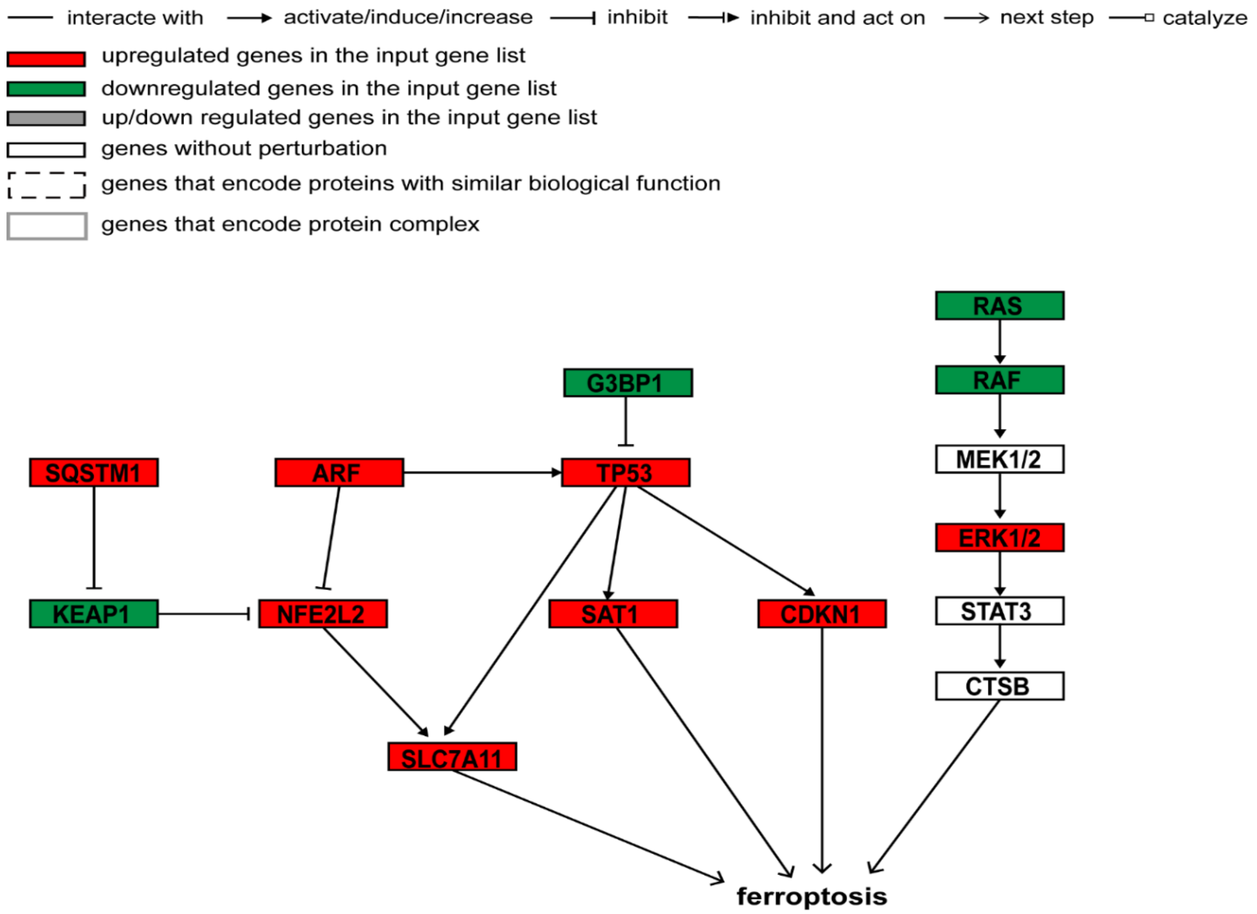

Supplementary Figure 5. Ferroptosis signaling pathway.

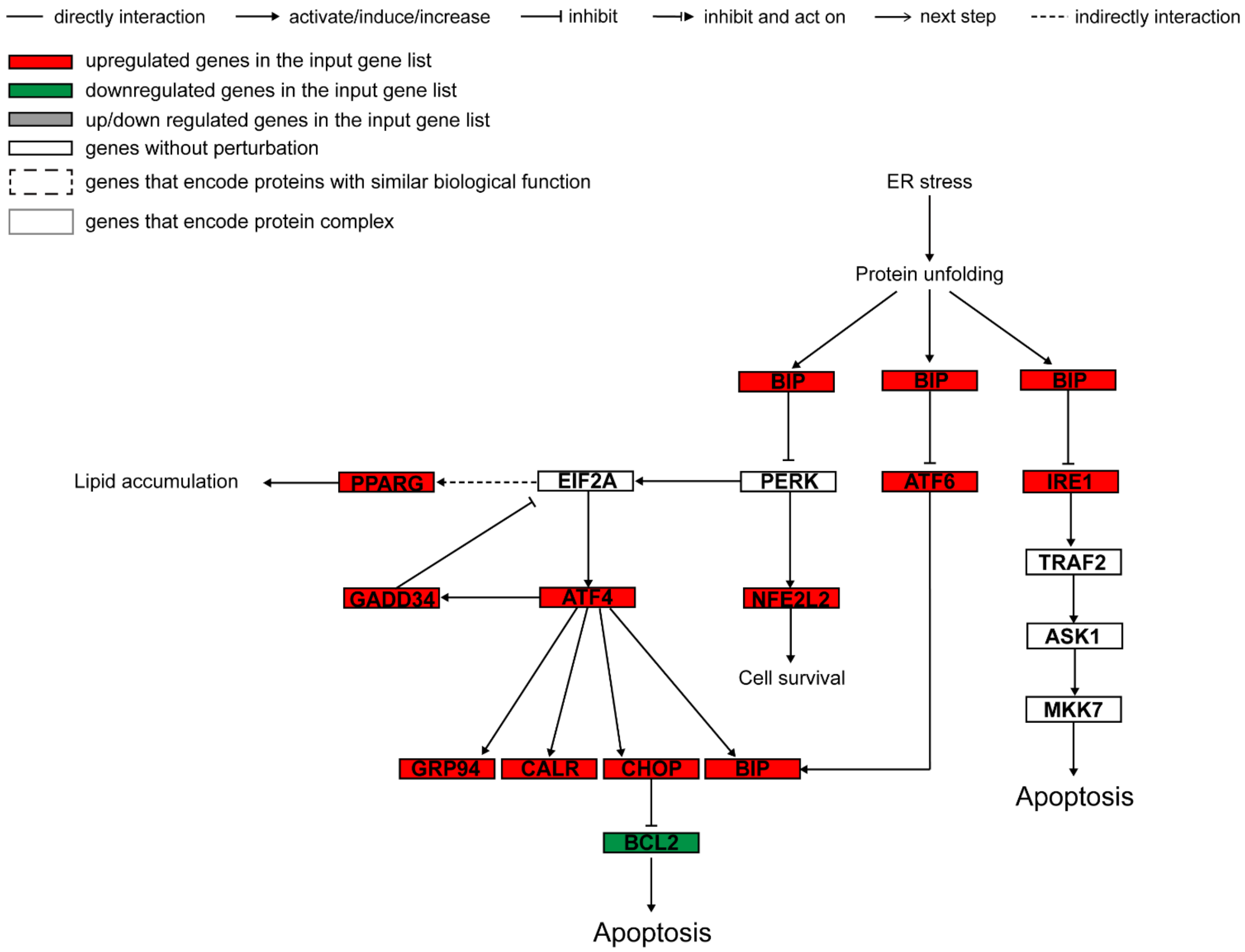

Supplementary Figure 6. Unfolded protein response.

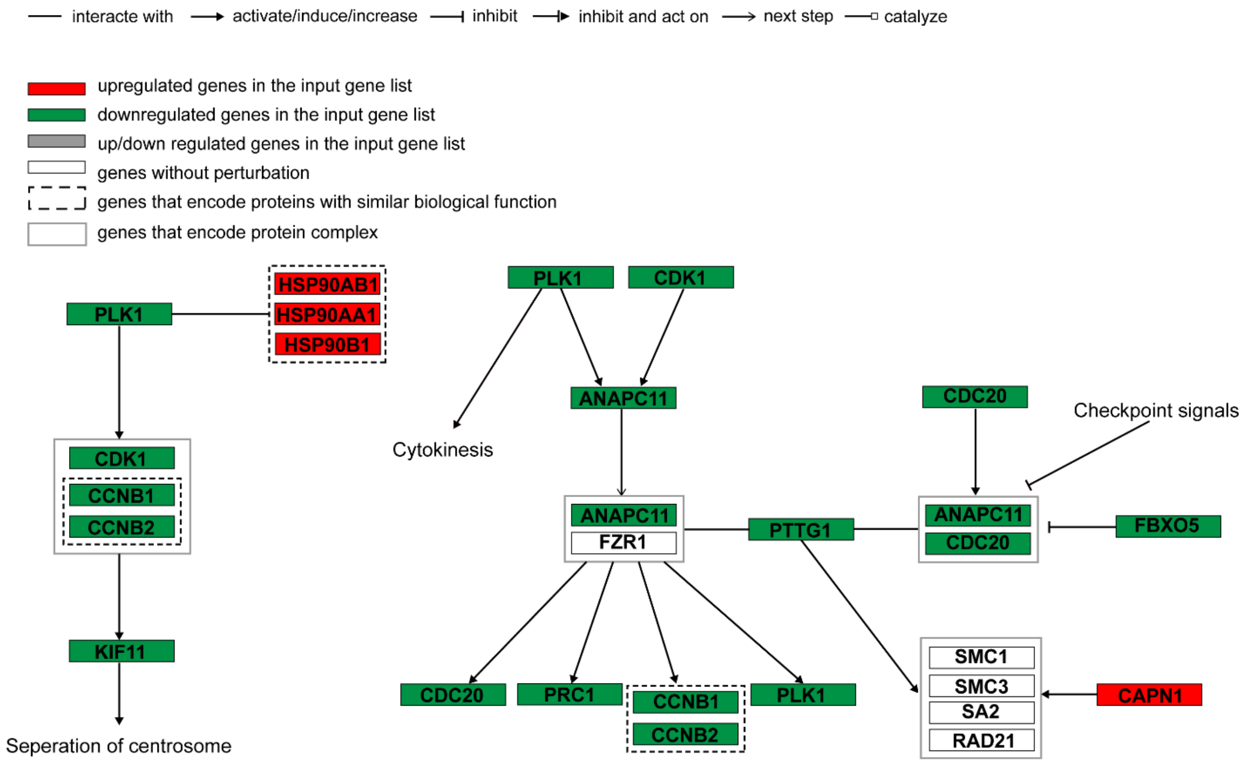

Supplementary Figure 7. Mitotic roles of polo-like kinase.

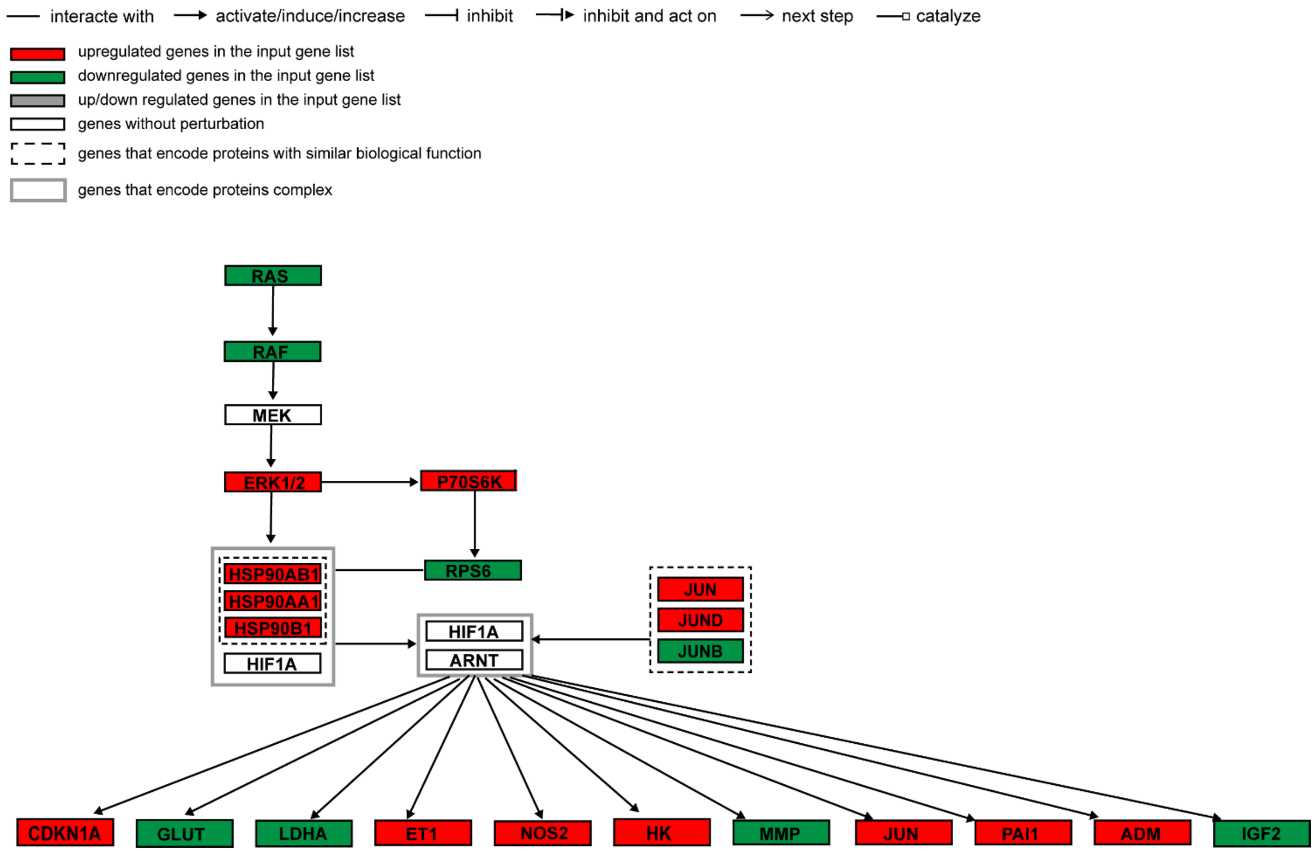

Supplementary Figure 8. HIF1α signaling.

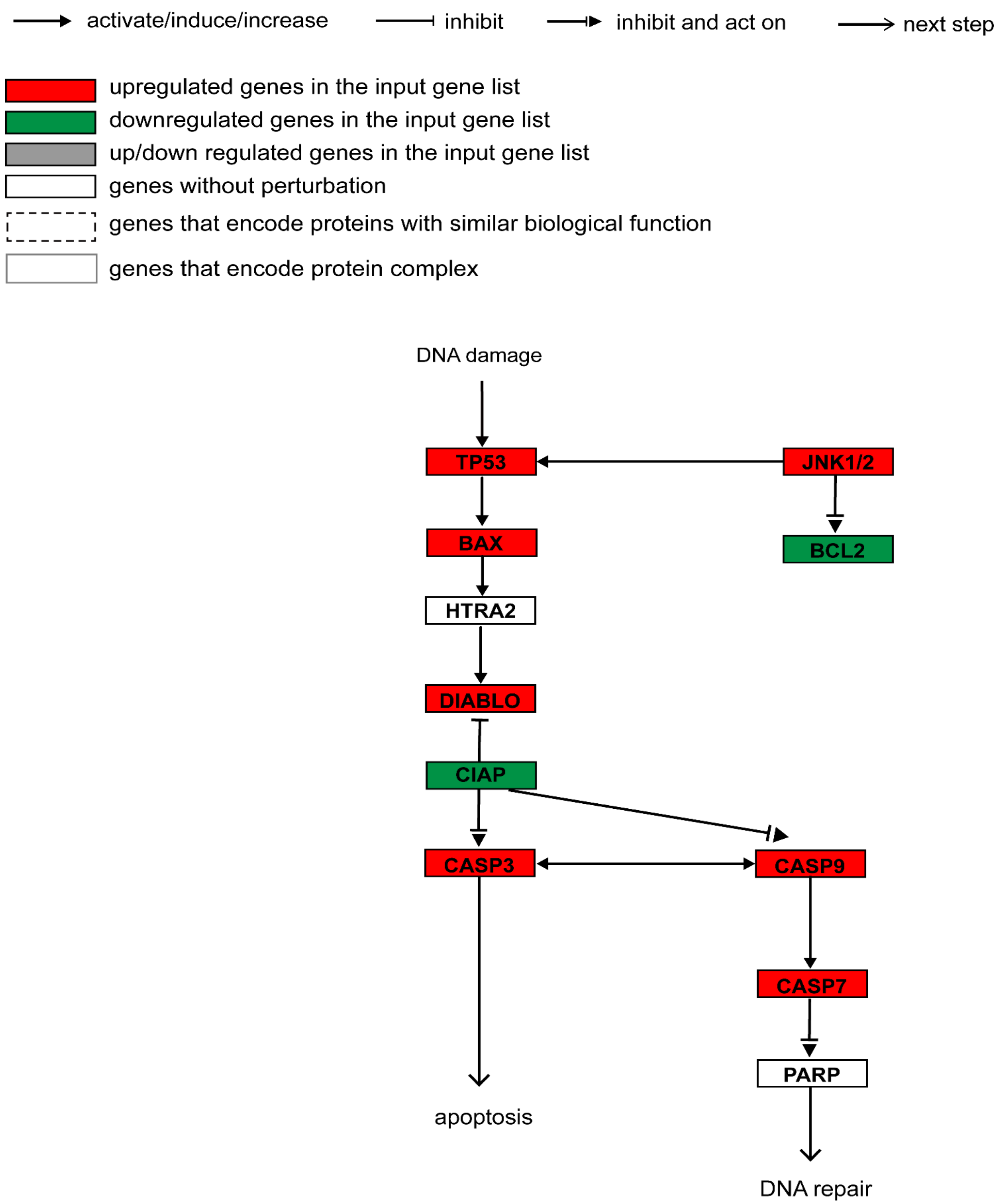

Supplementary Figure 9. Apoptosis signaling.

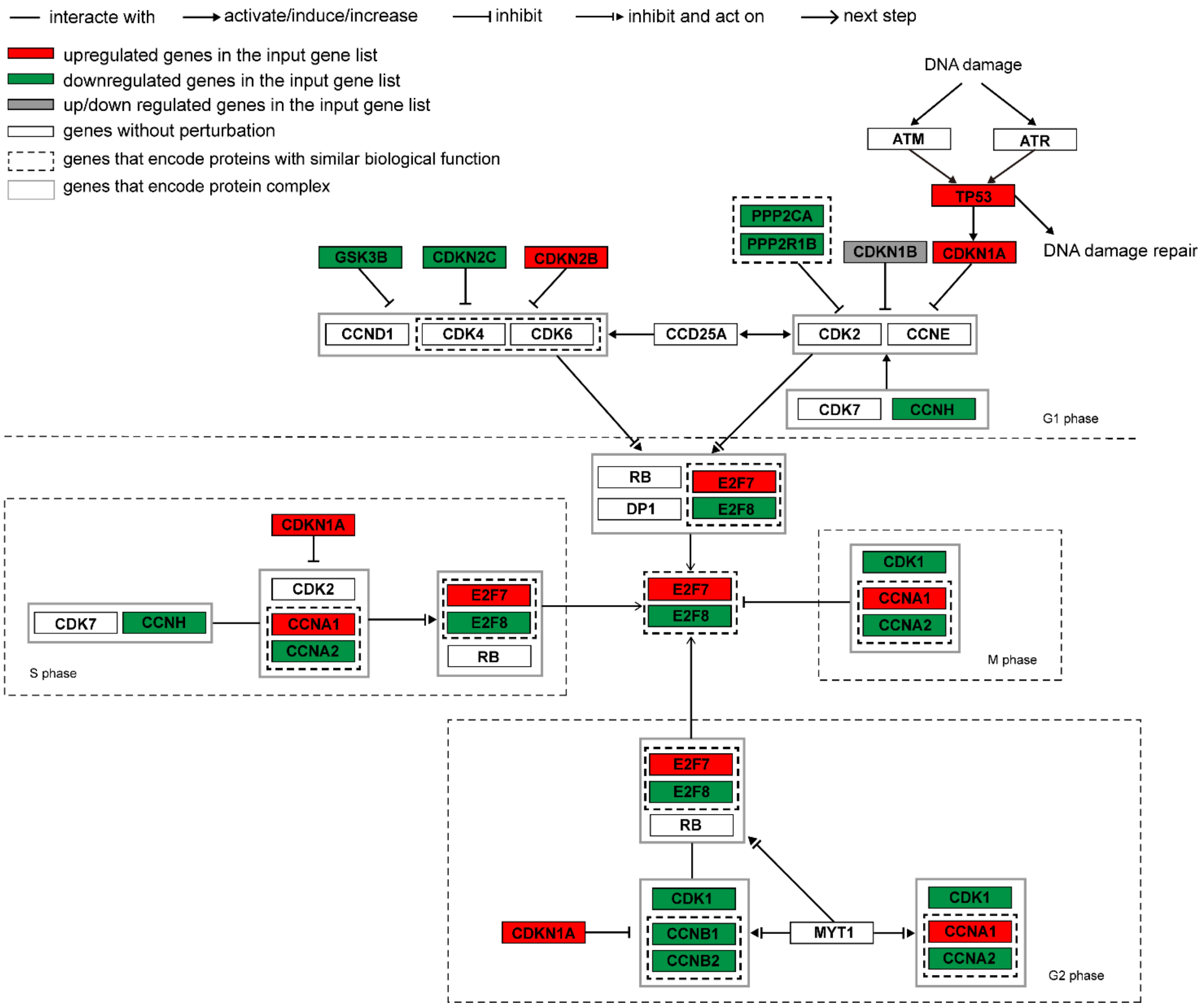

Supplementary Figure 10. Cyclins and cell cycle regulation

In supplementary figure 1—10, rectangles represent the perturbated genes in pathways. Rectangles in red represent upregulated genes in the gene list we input into IPA; green rectangles represent upregulated genes; gray rectangles represent up/down regulated genes (i.e., half of the studies showed up-regulation of gene expression by Cd while half reported down-regulation) and the white rectangles represent genes that relate to the perturbated genes but without perturbation by Cd themselves in the pathways; Rectangles surrounded by a dotted or solid border respectively represent genes encoding proteins with similar biological function and genes encoding protein complexes; arrows and lines represent the regulation direction of relationship between genes according to the Ingenuity Knowledge Base database.

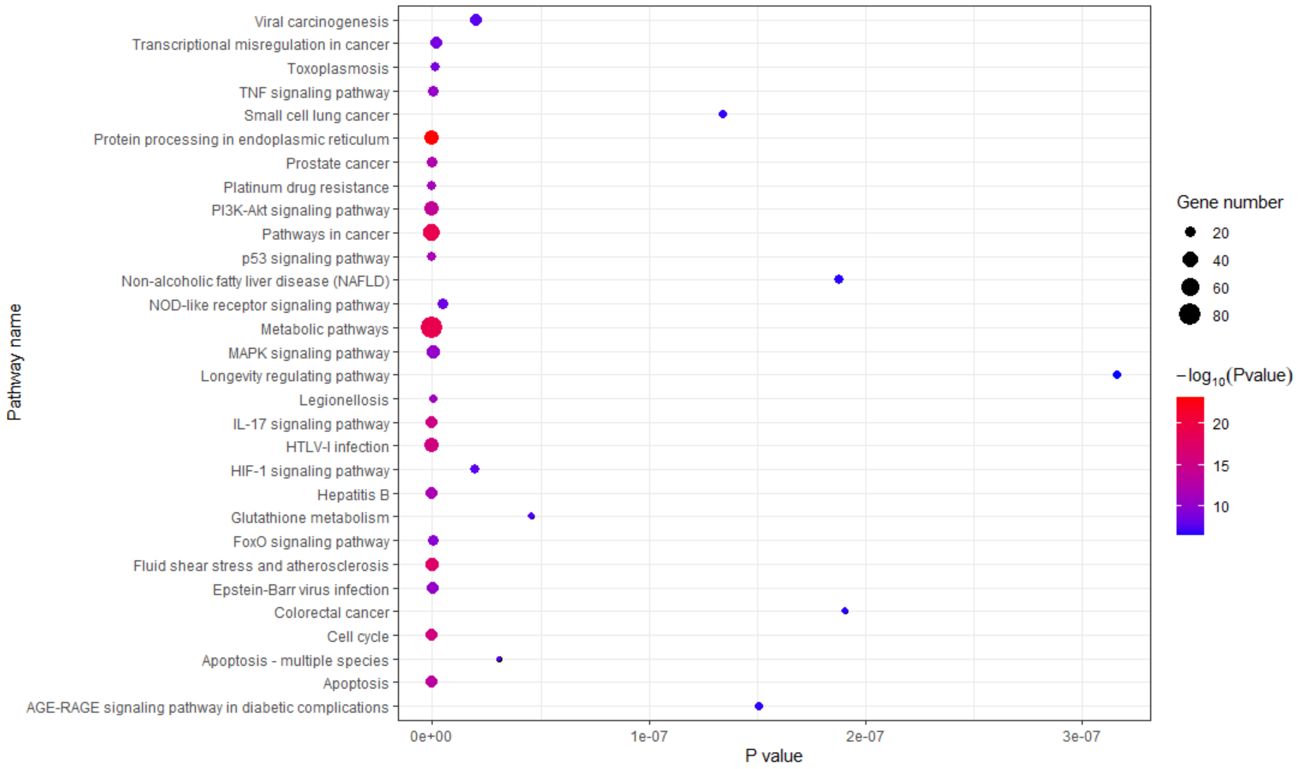

Supplementary Figure 11. Top 30 pathways generated by CTD.
